# Screening and identification of B-cell epitopes within envelope protein of tembusu virus

**DOI:** 10.1186/s12985-018-1052-1

**Published:** 2018-09-17

**Authors:** Dongmin Zhao, Kaikai Han, Xinmei Huang, Lijiao Zhang, Huili Wang, Na Liu, Yujie Tian, Qingtao Liu, Jing Yang, Yuzhuo Liu, Yin Li

**Affiliations:** 1 Institute of Veterinary Medicine, Jiangsu Academy of Agricultural Sciences, 50 Zhongling Street, Nanjing City, Jiangsu Province 210014 People’s Republic of China; 2 Key Laboratory of Veterinary Biological Engineering and Technology, Ministry of Agriculture, Nanjing, Jiangsu Province People’s Republic of China

**Keywords:** Tembusu virus, B-cell epitope, Envelope protein, Neutralization

## Abstract

**Background:**

Tembusu virus is a newly emerging flavivirus that caused egg-drop syndrome in ducks in China. TMUV envelope protein is a major structural protein locates at the surface of tembusu virus particle. During tembusu virus infection, envelope protein plays a pivotal role in induction of neutralizing antibody. However, B cell epitopes within envelope protein have not been well studied.

**Method:**

A series of 13 peptides derived from E protein of tembusu virus were synthesized and screened by Dot blot with tembusu virus-positive duck serum. Potential B-cell epitopes were respectively fused with GST tag and expressed in *E. coli*. The immunogenicity and protective efficiency of epitopes were assessed in ducks.

**Results:**

Dot blot assay identified the peptides P21 (amino acids 301–329), P23 (amino acids 369–387), P27 (amino acids 464–471) and P28 (amino acids 482–496) as potential B-cell epitopes within the envelope protein of tembusu virus. Immunization of prokaryotically expressed epitopes elicited specific antibodies in ducks and the specific antibody elicited by P21, P27 and P28 could neutralized tembusu virus. In addition, protective test suggested that P21 and P27 could completely protect immunized ducks from TMUV challenge.

**Conclusion:**

Four potential B cell epiotpes within tembusu virus envelope protein were identified and analyzed in vitro and in vivo. It was demonstrated that two of them (P21 and P27) could elicit neutralizing antibodies in ducks and offer complete protection against tembusu virus challenge. This findings will contribute to the development of epitope vaccine for tembusu virus prevention.

## Background

Tembusu virus (TMUV) is a newly emerging flavivirus that caused rapid egg drop and neurological symptoms in ducks in China since 2010. In egg-laying ducks, the TMUV infection lead to a decrease in egg production of 20% to 90% within 1 to 2 weeks post infection [1, 2]. Besides, affected ducks exhibited high fever, paralysis, ataxia, anorexia and diarrhea [3]. The infection and morbidity rates are up to 100%, with a mortality ranges from 5 to 30%, probably due to subsequent infections of bacteria [4]. The sudden outbreak and fast spread of TMUV in the major duck-producing provinces of China resulted in huge economic loss. Until now, in addition to ducks, other poultry, such as geese, chickens, pigeons and house sparrows can also be affected by TMUV. It has become one of the most important infectious diseases which threaten development of domestic poultry industry [5].

The TMUV, like other flavivirus, contains an approximately 11 kb single-stranded positive-single RNA genome that codes for a large polyprotein. The polyprotein is then cleaved into structural proteins and nonstructural proteins by proteases of TMUV and host cells [6]. The structural proteins include a single nucleocapsid protein (C), a liquid membrane protein (M) and the major envelope protein (E) [7]. In mature virus, E protein forms homodimer in head-to-tail conformation and assembles dense lattice on the surface of virion [8]. The flavivirus E protein mediates virus receptor binding and is a primary determinant of host range, cell tropism and virulence. Also, it plays an essential role in induction of neutralizing antibodies in the process of immune response [9, 10]. According to flavivirus convention, TMUV E protein can be divided into four domains, three of which form an N-terminal ectodomain that lies on the virion surface [11, 12]. Domain I (DI) is a structurally central amino terminal domain and acts as a bridge-like hinge linking the extended DII and DIII together [13]. Domain II (DII) contains two segments that project from loops of DI [1] and contributes to dimerization and membrane fusion [6]. Domain III (DIII) is located at the C-terminal of E ectodomain and has an immunoglobulin-like fold. DIII acts as the binding region for cellular receptor and is used as an antigen which can elicit neutralizing antibodies [14]. Following the DI/DII/DIII domains is the C-terminal domain IV which contains stem region and transmembrane domains. Domain IV anchors the ectodomain of E protein in TMUV membrane. Together with ectodomain of E protein, domain IV plays important roles in membrane fusion and mediates irreversible conformational changes in the process of fusion [15].

Virus infection elicits production of antibodies directed against epitopes within viral protein by triggering host humoral immunity [16]. Antibodies are considered to be critical for the inhibition of flavivirus infection in vivo, and this inhibitory effects are associated with neutralizing activity in vitro [17]. The neutralizing antibodies elicited by E protein inhibit the process of viral entry, including attachment between virus and host cell surface and fusion of viral and host membranes [7]. Studies of functional antibody and epitope mapping demonstrated that DI, DII and DIII of E protein are antigenic and possesses nonneutralizing or neutralizing B cell epitopes. DI predominantly contains type-specific nonneutralizing epitopes [18]. DII is composed of many cross-reactive epitopes which induces neutralizing and nonneutralizing monoclonal antibodies [19]. DIII contains multiple type- and subtype epitopes and elicit virus-neutralizing antibodies [18]. Therefore, identification of flavivirus B-cell epitopes is useful for peptide selection for epitope vaccines. Also, it is important for understanding virus-antibody interactions and development of specific serological diagnostic reagents [16].

Although numerous potential B-cell epitopes within the E proteins for other flaviviruses have been well defined, only a few epitopes in TMUV E protein have been ever identified [19, 20]. In this study, TMUV E protein was analyzed and the potential B-cell epitope candidates were predicted by computer bioinformatics software. Then thirteen dominant epitopes within TMUV E protein were synthesized and screened by Dot blot using anti-TMUV serum. Four selected epitopes were fused with GST tag and expressed in *E. coli*. These B-cell epitope fusion proteins were evaluated in ducks for their immunogenicity and protective efficiency against TMUV challenge.

## Methods

### Cells, viruses and antibodies

BHK-21 cells were maintained with RPMI 1640 containing 10% inactivated fetal calf serum (FCS) and incubated at 37 °C under an atmosphere of 5% CO_2_. TMUV JS804 was isolated by our laboratory and propagated in BHK-21 cells as previously described [21]. 50% Tissue Culture Infective Dose (TCID_50_) of TMUV JS804 was calculated using the Reed-Muench’s method [22]. Duck anti-TMUV serum was generated and maintained in our laboratory. Entire E protein was prokaryotically expressed and purified by Ni-NTA Agarose (Qiagen) in our laboratory [1]. Peroxidase-labeled goat anti-duck IgG was purchased from the KPL company, USA. BeyoECL Plus Kit was obtained from the Beyotime Institute of Biotechnology. Specific-pathogen-free (SPF) ducks were purchased from Harbin Veterinary Research Institute, China. The study was approved by the Animal Care and Use Committee of Jiangsu Province.

### Epitope prediction and synthesis

Computational analysis was conducted to analyze the sequence of entire TMUV E protein (501aa). The B cell epitopes prediction was performed using BCEPRED server (http://crdd.osdd.net/raghava/bcepred/). Thirteen potential B cell epitope peptides were synthesized and purified by Nanjing Genscript Biotechnology company. The purity of peptides were > 95%. The sequences and positions of these peptides were shown in Table 1.

**Table 1:** Sequences and positions of synthstic peptides

Peptide	Sequence	Location	Nucleotide sequence
P16	19-EWIDVVLEGGSCVTI-33	19–33	
P17	51-TELAVVRSYCYEPKVSDV-68	51–68	
P18	80-AHNPKATYAEYICKK-94	80–94	
P19	151-TYHNYSAQQSLKHAARFVITPKSPVYTA-178	151–178	
P20	245-AHATKQSVVALASQEGALHAALAGAIPVKYSG-276	245–276	
P21	301-TYPMCSNTFSLVKNPTDTGHGTVVVELSY-329	301–329	5’-ACCTACCCGATGTGTAGCAATACATTTTCCCTAGTGAAGAATCCTACCGACACTGGGCATGGCACTGTCGTGGTGGAATTGTCTTAT-3’
P22	351-PVGRLITVNPYVST-364	351–364	
P23	369-AKIMVEVEPPFGDSFILVG-387	369–387	5’-GCCAAGATAATGGTGGAAGTGGAACCTCCATTCGGGGATTCATTCATCTTAGTAGGA-3’
P24	430-SVGGVLTSIGKGIHQVFGSAFKS-452	430–452	
P25	475-NARDRSISM-483	475–483	
P26	392-QIRYQWHRSGS-402	392–402	
P27	464-MLGALLLW-471	464–471	5’-ATGTTGGGGGCACTGCTATTGTGG-3’
P28	482-SMTFLAVGGILVFLA-496	482–496	5’-TCTATGACTTTTCTAGCTGTAGGAGGAATTTTAGTCTTCCTGGCA-3’
Negative peptide	YIRTPACWD	Li et al., 2016a	

### Dot blot screening

The reactivity of predicted epitopes to anti-TMUV serum was determined by Dot blot as previously described [19]. Briefly, 1 μg of each synthesized epitope peptide was spotted on to a nitrocellulose membrane. The membrane was blocked by 5% BSA at 37 °C for 2 h. The membrane was washed three times with PBST and then incubated with duck anti-TMUV serum at 37 °C for 1 h, followed by incubation with peroxidase-labeled goat anti-duck IgG. The signal was detected using TMB Solution for Immunohistochemistry or Blotting (Beyotime) in accordance with the manufacturer’s protocol.

### Expression and purification of fusion peptides

Four peptides (P21, P23, P27, P28) which exhibited positive reactivity to anti-TMUV serum were expressed in *E. coli* BL21 as fusion proteins with GST tags. The gene fragment encoding each peptide was synthesized and subcloned into *Eco*R I and *Sal* I sites of bacterial expression vector pGEX-4 t-1 by Nanjing Genscript Biotechnology Company. The recombinant proteins were expressed and purified by Glutathione-agarose (Sigma). After purification, the proteins were detected by SDS-PAGE and confirmed by Western blot. Proteins were then quickly frozen and stored at -80 °C. The synthesized gene fragments are listed in Table 1.

### Duck immunization

9-day-old female SPF ducks were randomly divided into six groups (ten per group) and immunized i.m. The group 1, group 2, group 3, group 4 and group 5 respectively received 200 μg of purified recombinant P21, P23, P27, P28 and entire E protein emulsified with an equal volume of Freund’s complete adjuvant (Sigma), then followed by injection at 2 weeks afterwards with the protein emulsified in the Freund’s incomplete adjuvant. Group 6 were given 0.4 mL PBS as negative control in the same manner.

### Antibody assay by indirect ELISA

At two weeks after the last immunization, the sera were collected to perform antibody assay by indirect ELISA. Briefly, the 96-well ELISA plates were coated with purified TMUV at a concentration of 4 μg per well overnight at 4 °C. After blocking, the coated plates were washed with PBST three times, and incubated with 100 μL of sequentially diluted duck sera per well for 1 h at 37 °C. After washing three times with PBST, 100 μL of 1:1000 diluted peroxidase-labeled goat anti-duck IgG was add and incubated for 1 h at 37 °C. After three further washing, tetramethyl benzidine (TMB) substrate was added for six minutes at room temperature. The reaction was stopped with 2 mol/L H_2_SO_4_ and the OD450nm value of each well was read by using a BioTek microplate reader.

### Plaque reduction neutralization test

Plaque reduction neutralization test was performed to determine the neutralization ability of sera collected from immunized ducks as previously described [6]. Duck sera were heat-inactivated at 56 °C for 30 min and diluted 1:10 with RMPI 1640 containing 1% FBS. This inactivated sera were then two-fold serial diluted and incubated with an equal volume of TMUV (10^5^ TCID_50_) at 37 °C for 2 h. Then the virus-sera mixture was added in triplicate to BHK-21 monolayer cells. The remaining infectivity of virus was detected by plaque assay [23].

### Protective efficacy in ducks

All of the remaining ducks from six groups were injected i.m. with 10^4^ TCID_50_ of TMUV at 2 weeks after last boost. After injection, the steriled serum samples were collected daily. The serum samples were added to BHK-21 monolayer cells and incubated at 37 °C for 1 h. The inoculum was then removed followed by washing with PBS three times. The cells were covered by RPMI 1640 supplemented with 1% FCS and incubated at 37 °C for 72 h. Total RNA were extracted from cell cultures (QIAamp Viral RNA Mini Kit, Qiagen) and the viral nucleic acid was detected by qPCR as described above using previously described primers (Zhao et al., 2015b). The experiments were carried out in triplicate. If viral nucleic acid could be detected from serum-inoculated BHK-21 cells, the duck was considered to be infected (TMUV positive). The protection index (PI) was used to present protective efficacy of each epitope and calculated as follows: $\mathrm{PI}=\frac{\%\mathrm{TMUV}\ \mathrm{positive}\ \mathrm{in}\ \mathrm{negative}\ \mathrm{control}-\%\mathrm{TMUV}\ \mathrm{positive}\ \mathrm{in}\ \mathrm{immunized}\ \mathrm{group}}{\%\mathrm{TMUV}\ \mathrm{positive}\ \mathrm{in}\ \mathrm{negative}\ \mathrm{control}}\times 100$ [24].

### Statistical analysis

Statistical analysis of all data were performed with Statistics Package for Social Science Statistical (SPSS). Data are derived from at three replicates for each assay and presented as mean ± SD.

## Results

### Screening and identification of B cell epitopes by duck anti-TMUV serum

**Fig. 1:**
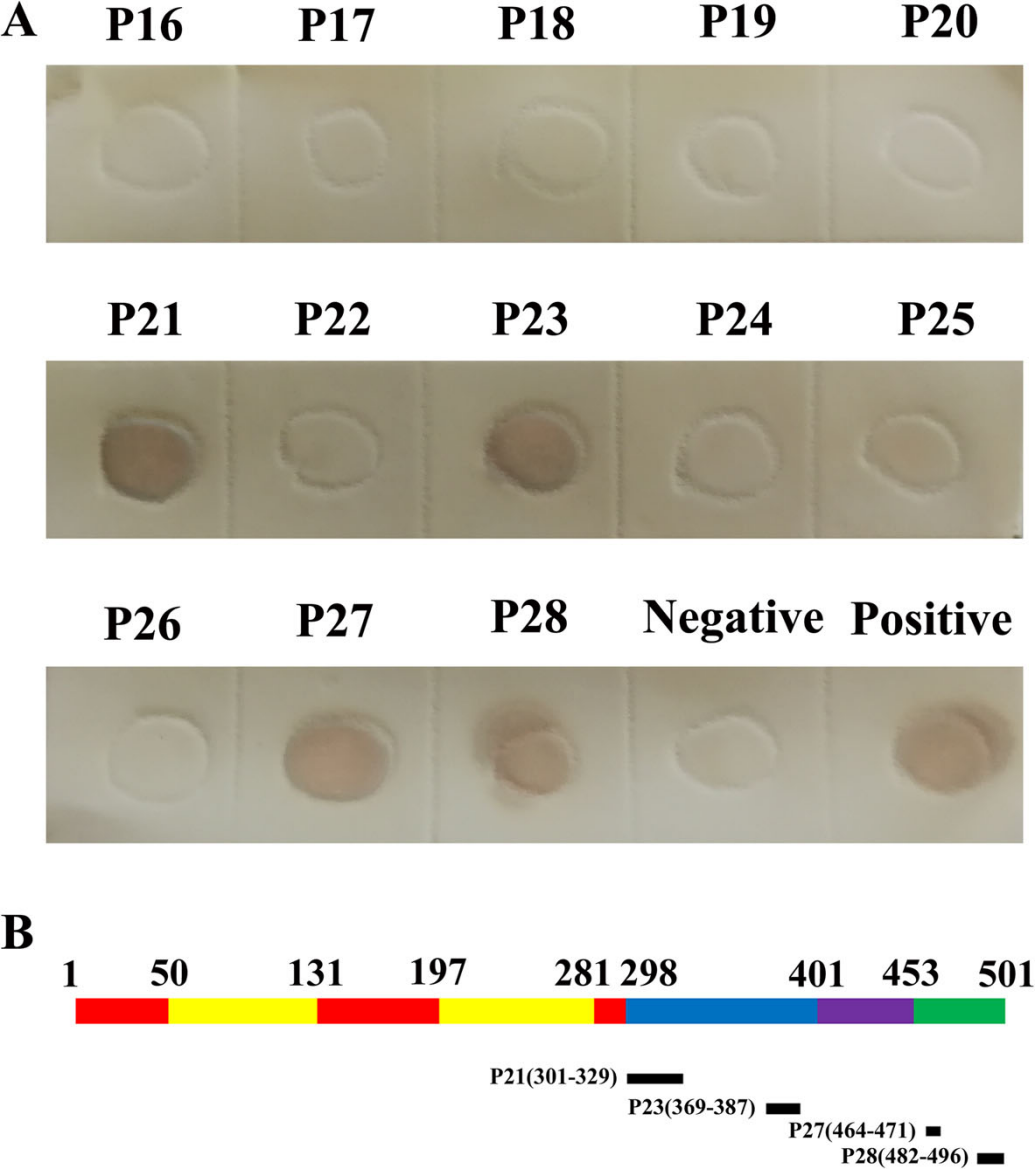
Identification of the B cell epitope by duck anti-TMUV serum. **a** Synthesized peptides were probed for reactivity with duck anti-TMUV serum by Dot-blot. Entire E protein and YIRTPACWD were used as positive and negative controls, respectively. **b** Structure of TMUV E protein showing the position of epitopes. E domains I, II and III were shown in red, yellow and blue. Stem and transmembrane domain were shown in purple and green

To screen the potential antigenic epitopes within E protein, TMUV E protein was analyzed by online software. Thirteen potential B cell epitope peptides were synthesized and tested whether these predicted peptides could be recognized by duck anti-TMUV serum. Dot blot showed that peptides P21, P23, P27 and P28 could react with the duck anti-TMUV serum, together with the entire E protein (Fig. 1a). The other nine peptides and negative control peptide could not bind with the anti-TMUV serum. Thus, the results suggested that these four potential B cell epitopes were involved in antibody binding, and their positions in E protein were indicated in Fig. 1b.

### Expression of GST-fused B cell epitope peptides

**Fig. 2:**
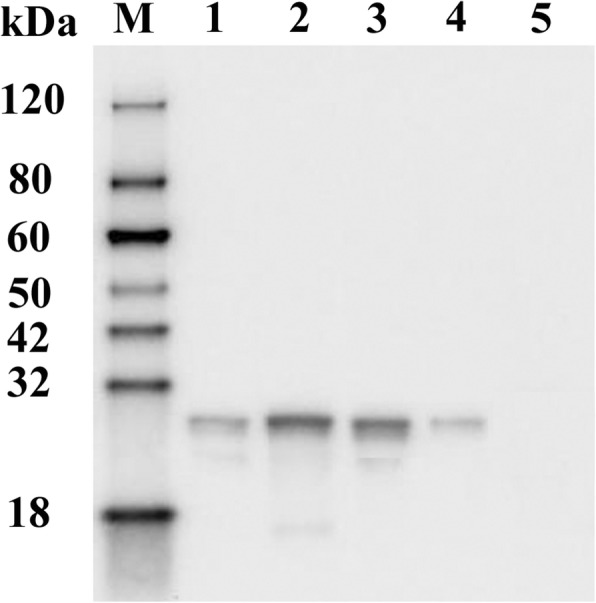
Detection of purified fusion proteins by Western blot using duck anti-TMUV serum. Lane M, molecular weight marker; Lane 1, P21; Lane 2, P23; Lane 3, P27; Lane 4, P28; Lane5, pGEX-4 t-1 vector

The encoding genes of four immunopositive peptides (P21, P23, P27, P28) were synthesized and successfully expressed in *E. coli*. All the GST-fused proteins were purified by Glutathione-agarose. Western blot showed that the purified fusion proteins could be recognized by anti-TMUV serum, confirming the reactivity of immunopositive peptides to anti-TMUV serum (Fig. 2).

### Immunogenicity of B cell epitopes

**Fig. 3:**
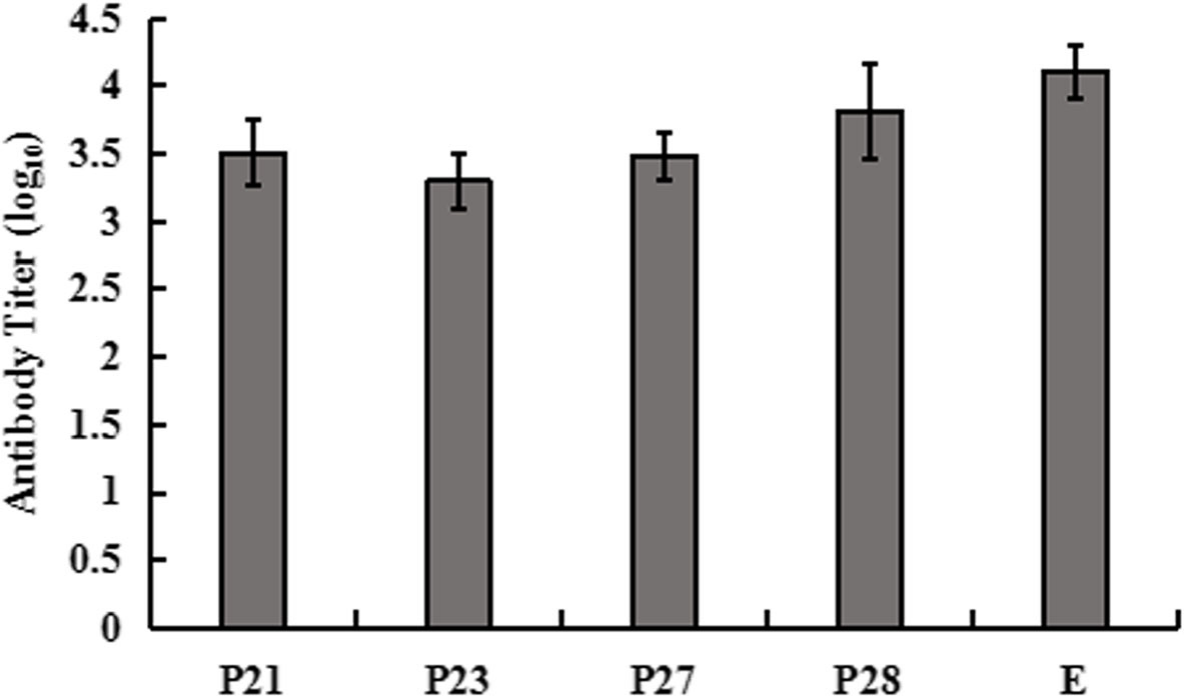
Antibody titer of ducks immunized with B cell epitopes. Groups of 9-day-old duck were immunized twice with P21, P23, P27, P28, entire E protein and PBS by intramuscular injection at 2 weeks interval. Serum samples were collected at 2 weeks after last boost and antibody titer was assessed by indirect ELISA

Specific antibodies elicited by fusion peptides were assessed by indirect ELISA. The purified TMUV was coated as a capture antigen. Serum samples were collected from each duck at two weeks after last boost. The results showed that duck immunized with fusion peptides generated a significant peptide-specific antibody response. It was observed that the level of antibody from ducks given the P28 was higher than that of ducks given with P21, P23 and P27. The level of entire E specific antibody was a littler higher than that of P28-specific antibody. These results suggested that fusion peptides stimulated significant TMUV-specific humoral immune response (Fig. 3).

### Neutralizing activity of anti-epitope antibodies

**Fig. 4:**
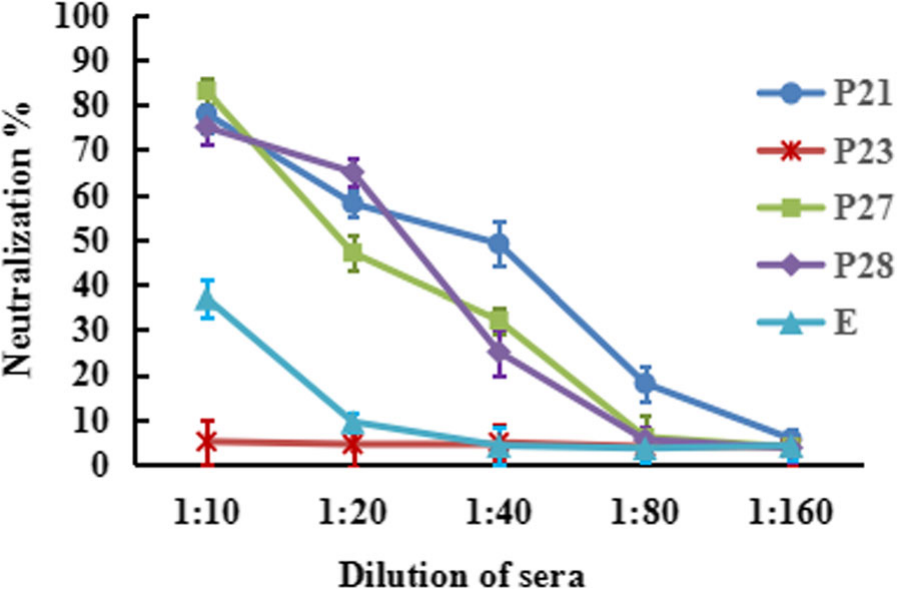
Neutralizing activity of anti-epitope antibodies against TMUV. Diluted sera were used to test the neutralizing activity against TMUV by plaque reduction neutralization assay. Data were presented from three independent experiments and statistic analysis was done with SPSS software

Various diluted serum samples were used to test the neutralizing activity against TMUV by plaque reduction neutralization test on BHK-21 cells (Fig. 4). The neutralizing response against TMUV was observed in sera from ducks immunized with P21, P27, P28 and entire E protein with neutralizing antibody titers of 1:80, 1:40, 1:40 and 1:10, respectively. In contrast, antibodies from ducks given the P23 did not show any neutralizing responses against TMUV, indicating that the antibodies induced by P23 could bind to TMUV but did not exhibit neutralizing activity.

### Protective efficacy against TMUV challenge in ducks

**Fig. 5:**
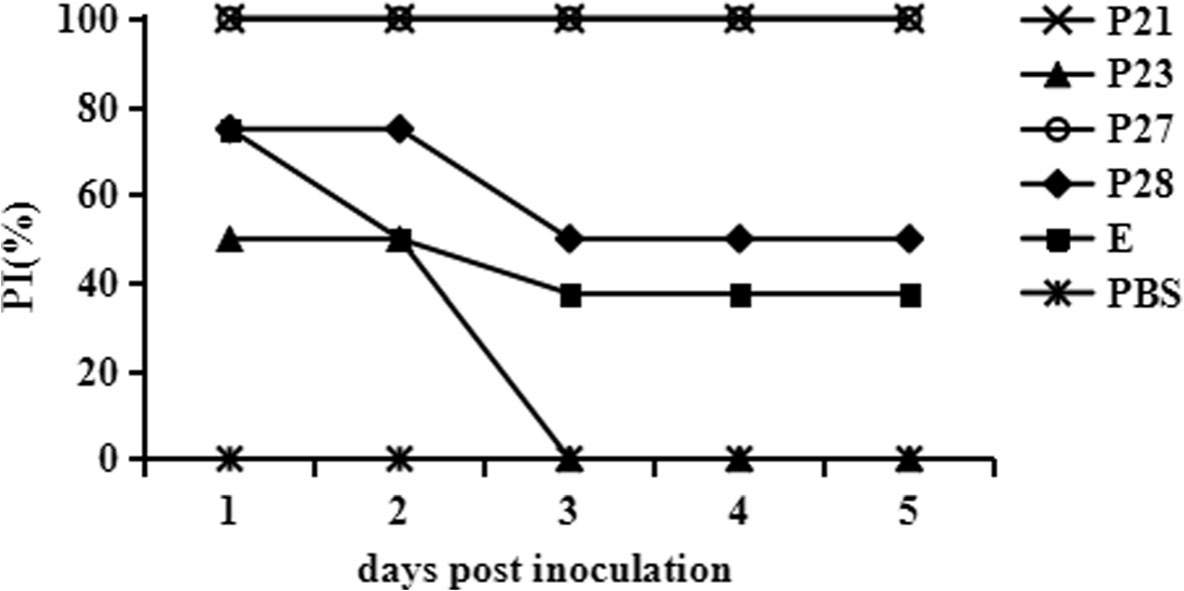
Protective efficacy against TMUV challenge in ducks. Six groups of ducks were inoculated intramuscularly with 10^4^ TCID_50_ of TMUV. Serum samples were collected daily and inoculated onto BHK-21 monolayer. After 3-day incubation, cell cultures were harvested for detection of TMUV by qPCR. The protection index (PI) was calculated to present protective efficacy of each epitope

To further investigate the protective efficacy of epitopes in ducks, all immunized ducks were intramuscularly challenged with 10^4^ TCID_50_ of TMUV at 2 weeks after last immunization. After the challenge, feed intake decrease and green-coloured faeces were found in PBS group and P23 immunized group from 1 day post inoculation. Additionally, appearances of neurological sign and weight loss were observed from 3 days after inoculation. The morbidity rates of PBS group and P23 immunized group were 100%. Four ducks of P28 immunized group and five ducks of entire E protein immunized group also showed clinical symptoms. It was further confirmed by viral nucleic acid detection. The rates of morbidity were 50% (4/8) and 62.5% (5/8), resulting in PI of 50% and 37.5% respectively. In contrast, ducks immunized with P21 and P27 did not exhibit clinical symptoms and TMUV positive during the whole observation period (5 days), indicating complete (PI = 100%) protection against TMUV challenge (Fig. 5).

## Discussion

The newly outbreak and wide spread of TMUV severely threatens several domestic poultry species and resulted in serious economic loss. With no specific treatment against TMUV infection, vaccine immunization is considered as the most effective measures to fight against the serious infection in domestic poultry industry [24]. Up to now, despite inactivated and attenuated live vaccines have been licensed by Chinese ministry of agriculture, the problem of antibody dependent enhancement caused by inactivated virus and the biosecurity concerns with attenuated live virus vaccine are highly arguable [1]. Therefore, precedence is given to developing more security, effective and affordable vaccine against TMUV. Peptide vaccine containing specific epitope capable of stimulating humoral and cellular immune reactions represents an alternative strategy [25]. An earlier study showed that recombinant multi-epitope peptide from Japanese encephalitis virus could elicit humoral and cellular immune responses and protected mice from lethal Japanese encephalitis virus challenge [26]. Furthermore, peptide vaccine can be used to induce specific immunity against selected epitope(s) while avoiding allergenic and/or reactogenic complications [6]. In this paper, we focused on the screening and identifying B cell epitopes within TMUV E protein.

B cell epitopes are classified as either continuous or discontinuous. The continuous (also called linear) epitope is a consecutive fragment from the protein sequence, and discontinuous epitope is composed of several fragment scattered on the protein sequence, which form secondary, tertiary or quaternary structure [27]. But the distinction between continuous and discontinuous epitope is vague. The discontinuous epitope consists of several consecutive amino acids that can be considered as continuous epitopes [28]. Moreover, since TMUV is a newly emerging virus, the crystal structure of E protein is not determined and accurate protein structure data are limited. In such a scenario, we analyzed sequence of TMUV E protein and predicted potential continuous epitopes using bioinformatics software.

In previous studies, peptides were synthesized or expressed to identify the B cell epitopes by three widely used methods, Western blot, ELISA or Dot blot [29–31]. Dot blot is a simplification of Western blot method, with the advantage of simple and effective. It can be also used to explore the binding availability of peptide against the antibody. Li et al. identified a new broadly cross-reactive epitope within domain III of TMUV E protein by Dot blot [19]. However, the results of Dot blot offers no information on the molecular size of the target peptide. In this study, Dot blot assay indicated that four peptides (P21, P23, P27 and P28) were recognized by anti-TMUV serum. Genes encoding these four epitopes were synthesized and cloned to pGEX-4 t-1 vector, then expressed as GST-fused proteins in *E. coli*, respectively. Subsequently, the immunogenicities of all the four epitopes was evaluated in ducks.

The DIII domain was reported to elicit strong neutralizing response against flaviviruses [6] and the P21 representing amino acid position 301–329 is located within this domain. The antibody elicited by P21 was able to neutralize TMUV with a titre of 1:80. In addition, immunization of P21 in ducks induced complete protection against TMUV challenge, indicating that P21 is a neutralizing epitope on DIII and might be attractive for development of epitope vaccine.

Although P23 also located in DIII, plaque reduction neutralization test showed that antibody elicited by P23 did not neutralize TMUV. However, P23 contained a motif, 374-EVEPPFG-380, which was a immunodominant and cross-reactive epitope (EXE/DPPFG) that is highly conserved in most flaviviruses [4], including west nile, zika, dengue, Japanese encephalitis and yellow fever viruses. This highly conserved motif may be useful in broad detection of TMUV and other flaviviruses. In contrast, the nonneutralizing epitope P23 can be deleted in future epitope vaccine development to reduce induction of nonneutralizing antibody and limit the formation of nonneutralized antibody complexes.

A previous study proved that the transmembrane domain of E protein displays either low B cell antigenicity or antigenically inert and no B cell epitope was identified in this region [32, 33]; however, these studies were inconsistent with another research that transmembrane domain of Japanese encephalitis viruses contains a highly reactive B cell epitope [34]. In this study, epitopes P27 and P28 located in transmembrane domain of E protein and elicited neutralizing antibody against TMUV. In ducks, P27 immunization provided fully protection against subsequent TMUV challenge, but only 50% P28 immunized ducks could be protected from challenge. In flavivirus virion, as was known to all that the C protein was not exposed on the surface of viral particles, however, several studies have reported that C protein stimulated B-cells during viral infection and the epitopes on C protein have been defined using immune sera from individuals infected with virus [35–37]. The most likely mechanism by which C protein can elicit specific antibody responses is by way of structural dynamic “breathing” (translocation of internal proteins to the exterior viral surface) [38]. It was likely that C protein was transiently exposed to the viral surface via viral breathing. We speculated that P27 and P28 may induce antibodies by a similar mechanism. Thus, the antibodies that were induced by P27 and P28 involved in protective responses possibly through the formation of immune complexes, complement activation or antibody-dependent cellular cytotoxicity [39]. Nevertheless, it is something worth investigating further.

## Conclusion

We first identified four potential B cell epiotpes within TMUV E protein and analyzed their protection efficacy in ducks. It was demonstrated that two of them (P21 and P27) could elicit neutralizing antibodies in ducks and offer complete protection against TMUV challenge. Our findings provide valuable information for understanding antigenic structure of TMUV E protein and useful epitopes for the development of vaccine to prevent TMUV infection.
